# A Novel Missense Mutation of c.965C>T (p.Ala322Val) in the Human *GALNS* Gene Results in Severe Mucopolysaccharidosis Type IVA

**DOI:** 10.30699/ijp.2024.2025376.3278

**Published:** 2025-01-10

**Authors:** Moeinadin Safavi, Aria Setoodeh, Mahdiieh Ghoddoosi

**Affiliations:** 1 *Molecular Genetic Pathology Lab, Children’s Medical Center, Tehran University of Medical Sciences, Tehran, Iran*; 2 *Department of Endocrinology, Children’s Medical Center, Tehran University of Medical Sciences, Tehran, Iran*; 3 *Department of Pathology, Qom University of Medical Sciences. Qom, Iran*

**Dear Editor,**

Mucopolysaccharidosis IVA (Morquio syndrome) is a cause of morbidity in early or late childhood, depending on disease severity. It is an autosomal recessive disorder characterized by short stature and multiple skeletal abnormalities. A deficiency of N-acetylgalactosamine-6-sulfate sulfatase (GALNS) activity, due to mutations in the *GALNS* gene, leads to the accumulation of keratan sulfate, which destroys cartilage tissue before epiphyseal closure (1). Moreover, respiratory compromise, as well as cardiac, ocular, dental, hearing, and neurologic impairments, are consequences of disease progression (2, 3).

Early and accurate diagnosis, based on the detection of low enzyme activity or through molecular genetic testing, may facilitate early intervention and improve the quality of life for affected children (4–6).

**Fig. 1:**
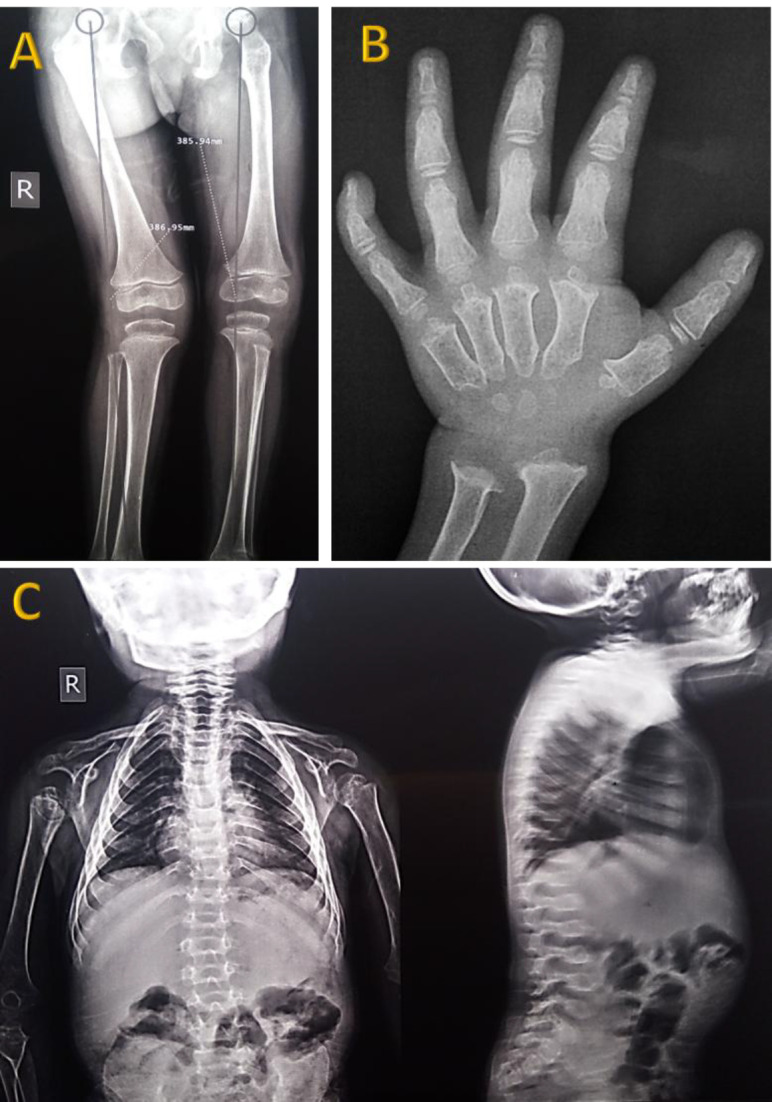
Skeletal abnormalities in our case of mucopolysaccharidosis type IVA. X- ray examination shows genu valgus (A), metaphyseal dysplasia, proximal metacarpal beaking, ulnar deviation of radius and ulna (B) and kyphoscoliosis (C).

To date, the number of identified mutations in this disease continues to increase. We aim to introduce a novel missense point mutation in the *GALNS* gene that is associated with a severe form of MPS IVA. This mutation was detected in a three-year-old girl, born of consanguineous parents, who presented with genu valgus, pectus carinatum, cupping of the wrists, ulnar deviation of the radius and ulna, and kyphoscoliosis (Figure 1). The Berry spot test of the urine was positive for 

**Fig. 2:**
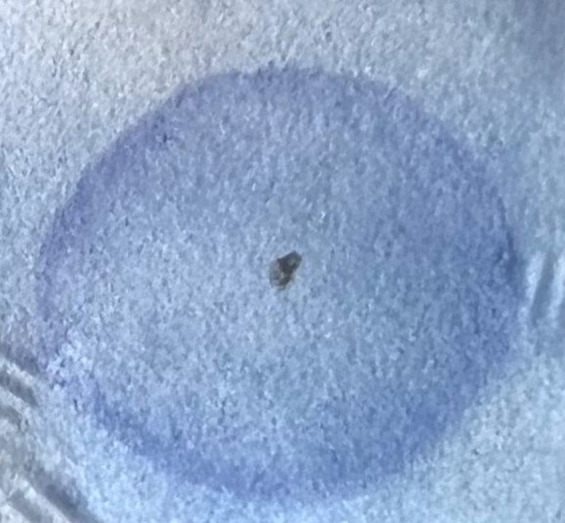
Positive urine Berry spot test in our case.

Mucopolysaccharides (Figure 2). Cell blood count, eythrocyte sedimentation rate, fasting blood sugar, creatine phosphokinase, calcium, phosphorous, alkaline phosphatase, amylase, and 25-OH-vitamin D levels were all within the normal range.

Whole exome sequencing, performed using the Illumina Novaseq 6000 system (151 bp paired-ends read, depth of coverage: 100x) and compared with the published human genome build (UCSC hg19 reference sequence), revealed a c.965C>T (p.Ala322Val) missense variant in both alleles of the *GALNS* gene. This variant is classified as a likely pathogenic variant according to the American College of Medical Genetics (ACMG) guidelines, which is consistent with a molecular diagnosis of Mucopolysaccharidosis IVA. 

In conclusion, this novel mutation is associated with severe form of MPS IVA. Early detection of this genetic alteration in a suspected patient, may help in in early treatment with enzyme, prediction of disease severity and provide a better quality of life by early intervention.
